# The dynamic nature of patient engagement within a Canadian patient‐oriented kidney health research network: Perspectives of researchers and patient partners

**DOI:** 10.1111/hex.13716

**Published:** 2023-01-27

**Authors:** Meghan J. Elliott, Tamara L. McCarron, Kara Schick‐Makaroff, Leah Getchell, Braden Manns, Nicolas Fernandez

**Affiliations:** ^1^ Department of Medicine University of Calgary Calgary Alberta Canada; ^2^ Department of Community Health Sciences University of Calgary Calgary Alberta Canada; ^3^ O′Brien Institute for Public Health University of Calgary Calgary Alberta Canada; ^4^ Faculty of Nursing University of Alberta Edmonton Alberta Canada; ^5^ CanSOLVE CKD Network Patient Partner Vancouver BC Canada; ^6^ Department of Family Medicine and Emergency Medicine Université de Montréal Quebec Montreal Canada

**Keywords:** chronic kidney disease, patient engagement, patient‐oriented research, qualitative research

## Abstract

**Introduction:**

Canadians Seeking Solutions and Innovations to Overcome Chronic Kidney Disease (Can‐SOLVE CKD) is a pan‐Canadian health research network that engages patients as partners across 18 unique projects and core infrastructure. In this qualitative study, we explored how research teams integrated patient partners into network research activities to inform our patient engagement approach.

**Methods:**

To capture a breadth of perspectives, this qualitative descriptive study purposively sampled researchers and patient partners across 18 network research teams. We conducted 4 focus groups (2 patients and 2 researchers; *n* = 26) and 28 individual telephone interviews (*n* = 12 patient partners; *n* = 16 researchers). Transcripts were coded in duplicate, and themes were developed through an inductive, thematic analysis approach.

**Results:**

We included 24 patient partners and 24 researchers from 17 of the 18 projects and all core committees within the network. Overarching concepts relate participants' initial impressions and uncertainty about patient engagement to an evolving appreciation of its value, impact and sustainability. We identified four themes with subthemes that characterized the dynamic nature of patient engagement and how participants integrated patients across network initiatives: (1) Reinforcing a shared purpose (*learning together, collective commitment, evolving attitudes*); (2) Fostering a culture of responsive and innovative research (*accessible supports, strengthened process and product*); (3) Aligning priorities, goals and needs (*amenability to patient involvement, mutually productive relationships, harmonizing expectations*); (4) Building a path to sustainability (*value creation, capacity building, sustaining knowledge use*).

**Conclusions:**

Our findings demonstrate the dynamic and adaptive processes related to patient engagement within a national, patient‐oriented kidney health research network. Optimization of support structures and capacity are key factors to promote sustainability of engagement processes within and beyond the network.

**Patient or Public Contribution:**

This project was conceived in collaboration with a Can‐SOLVE CKD patient partner (N. F.), with lived experience of kidney failure. He also co‐designed the study′s protocol, led focus groups and researcher interviews, and contributed to data analysis. L. G. has lived experience as a caregiver for a person with CKD and facilitated patient partner focus groups. The patient partners, both of whom are listed authors, provided important insights that shaped our interpretation and presentation of study findings.

## 1 INTRODUCTION

Chronic kidney disease (CKD), defined as persistent structural or functional abnormalities of the kidney, is a significant public health concern that affects approximately 10% of the global population.^1^, ^2^ Care for persons with CKD is complex in that disease manifestations are varied and treatment options encompass both strategies to slow progression and manage complications.^3^ In addition to its high associated morbidity, mortality and healthcare costs,^4^ CKD management is complicated by a lack of high‐quality evidence guiding clinical care.^5^, ^6^, ^7^ Moreover, areas of highest kidney health research activity do not necessarily align with the priorities of health system users, including patients, whose involvement as partners in research has been increasingly sought to enhance research relevance and application in practice.^8^

The experience of living with a condition such as CKD means that patients, and those who care for them, hold valuable insight into how service delivery could be optimized to meet their needs.^9^ Alongside international strategies that support purposeful and meaningful involvement of patients and the public in health research, such as the Patient‐Centered Outcomes Research Institute (PCORI) in the United States^10^ and National Institute for Health Research (NIHR) Centre for Engagement and Dissemination (formerly INVOLVE) in the United Kingdom,^11^ Canada's Strategy for Patient‐Oriented Research (SPOR) was launched to provide support for patient‐oriented research; that is, health research that engages patients as partners, focuses on patient‐identified priorities and enhances healthcare delivery and outcomes.^12^, ^13^ The Canadians Seeking Solutions and Innovations to Overcome Chronic Kidney Disease (Can‐SOLVE CKD) Network is one of five pan‐Canadian chronic disease patient‐oriented research networks supported through SPOR. The network's mandate is to overcome challenges to uptake of research evidence into practice through engagement with knowledge users (i.e., patients, caregivers, clinicians, policymakers) across the research cycle, from inception to implementation of findings.^14^ The active engagement of patients as partners in Can‐SOLVE CKD reflects a shift from the traditional view of patients as research subjects toward a collaborative model of co‐production of relevant, useable health innovation.

Several frameworks for patient and public involvement in research offer guidance on engagement approaches and practical strategies to promote meaningful collaboration, typically related to individual or smaller‐scale projects.^13^, ^15^, ^16^, ^17^ However, the broad scope of initiatives across Can‐SOLVE CKD meant that a single, uniform approach to patient engagement was neither feasible nor desirable. As diverse network teams operationalized and adapted engagement resources in response to their unique needs, it was unclear how patient partners could or should engage with researchers in their mutual pursuit of network objectives. Attempts to understand the perspectives of patients and researchers in this context are critical for establishing meaningful engagement processes and productive research partnerships.^18^ With an appreciation for the dynamic nature of patient engagement within Can‐SOLVE CKD, the purpose of this qualitative study was to explore how the network integrated patient partners into a wide range of research activities and initiatives to inform engagement structures and supports.

## 2 METHODS

### 2.1 Study Design and setting

The Can‐SOLVE CKD network encompasses 18 unique, multidisciplinary research projects spanning kidney health populations (i.e., nondialysis CKD, kidney failure, transplantation, paediatrics) and research pillars (i.e., 1 basic science, 4 translational and 13 clinical projects). Infrastructure to support patient engagement, knowledge translation and capacity building was provided through several committees centred around patients (e.g., Patient Governance Circle,^19^ Indigenous Peoples' Engagement & Research Council [IPERC]^20^). Over 50 patient partners and 130 researchers from across Canada have contributed to network research and governance.

The network referred to existing patient engagement resources initially to establish patient co‐leadership, team member roles and expected contributions across projects; more formal guidance documents and reporting processes were developed after the first year. In this qualitative descriptive study,^21^, ^22^ we outline how these processes took shape. We adhered to the consolidated criteria for reporting qualitative research^23^ (COREQ) and to the ethical conditions outlined by the health research ethics boards from the University of Calgary (REB18‐0131) and University of Montreal (#17‐138‐CERES‐D).

### 2.2 Participant recruitment

We employed a purposive, maximum variation sampling approach to identify and invite eligible individuals from Can‐SOLVE CKD to participate in this study. This sampling approach enabled selection of information‐rich individuals to characterize unique experiences and central themes.^24^ Any adult (≥18 years) patient partner or researcher within the network was eligible, although we aimed to sample across stakeholder roles and types of engagement activities to reflect a breadth of perspectives. This included deliberate sampling of patient partners and researchers with various roles on research project teams (i.e., project co‐lead, research advisor and/or study conduct [for patient partners]; principal investigator, co‐investigator or research support staff [for researchers]) and/or core network committees (i.e., co‐chair or member of the Training and Mentorship Committee, Patient Governance Circle, IPERC and/or Knowledge Translation Committee). The network considers a ‘patient’ as someone who has personal experience living with kidney disease of any type or stage, informal caregivers, such as family or friends, and living kidney donors. Eligible researchers included principal investigators, research staff and/or research trainees on project teams. Many researchers were also healthcare professionals with experience in care delivery to persons with CKD.

### 2.3 Data collection

Data were collected through focus groups and semistructured interviews. The use of both focus groups and interviews in a study is a form of methods triangulation, whereby the data arising from group interaction and discussion can complement and enhance that obtained through in‐depth, reflective interviews.^25^ Focus groups were conducted during an annual network meeting held 2 years into the network's 5‐year funding term and lasted 90 min. Each focus group was comprised of either patient partners or researchers to allow for in‐depth discussion, manage potential power differentials and enable expression of differing viewpoints in a safe setting. An observer took field notes to supplement the recordings and track important nonverbal communication or actions by participants. Individual interviews were conducted by telephone or videoconferencing to expand on issues raised during focus groups and other experiences related to research engagement. Interviews were conducted by one investigator and lasted 40–60 min. N. F. conducted patient partner interviews and M. J. E. conducted researcher interviews to minimize unfavourable dynamics, except in instances of perceived conflict or pre‐existing relationships. The question guides were developed by investigators based on familiarity with the engagement literature to explore perceived roles, experiences and challenges of engaging across projects/disciplines in kidney health research (see Supporting Information). Interviews and focus groups were audio‐recorded, transcribed verbatim and uploaded to NVivo 12 (QSR International Pty.) to facilitate data organization and retrieval. Demographic and network‐related information were collected to characterize our sample and inform our purposive sampling approach.

### 2.4 Data analysis

Data collection and analysis took place concurrently so that emerging concepts could be explored in subsequent interviews. Two investigators (N. F., M. J. E.) inductively analysed de‐identified transcripts using thematic analysis.^26^, ^27^ Through a systematic process, investigators first independently reviewed transcripts, noting initial impressions and developed a preliminary coding framework. This was refined through coding 10 transcripts (5 patient partners; 5 researchers), discussing code definitions and scope and then systematically applying codes to remaining transcripts. Coded extracts and relationships between concepts were then examined to develop themes based on meaningful clusters. Emerging themes were verified in relation to the data set for rigour and discussed among the broader research team to ensure consistency, credibility and relevance of findings. The comprehensiveness of our data set allowed us to develop subthemes that encompass important yet distinct concepts comprising each main theme. We presented preliminary findings at a subsequent Can‐SOLVE CKD Network annual general meeting that most study participants attended, and incorporated audience feedback and suggestions into our final thematic reporting. We maintained an audit trail of all analytic steps.

## 3 RESULTS

Forty‐eight individuals from across Canada participated in this study. We conducted 2 patient partner and 2 researcher focus groups (each with 4–10 individuals) and 28 interviews (12 patient partners; 16 researchers). Six patient partners participated in both an interview and focus group. Participants were represented on 17 of the 18 network projects and all core network committees. Of 24 participating patient partners, 14 contributed to 3 or more research project teams and 9 sat on more than 1 core network committee. Patient partner responsibilities and expected commitments varied across projects and committees, including attending regular meetings, providing input on study processes and materials, co‐developing tools, engaging with communities and disseminating findings. Of the 24 participating researchers, 17 contributed to only one research project team and 4 sat on at least 1 committee. Demographic characteristics for patient partner and researcher participants are provided in Tables 1 and 2, respectively.

**Table 1:** Characteristics of participating patient partners (*n* = 24).

Characteristic	Number
Sex	
Female	16
Male	7
Prefer not to answer	1
Age (year)	
<40	5
40–64	15
≥65	4
Location of residence	
Western Canada	15
Ontario	8
Quebec	0
Eastern Canada	1
Territories	0
Duration in Can‐SOLVE CKD Network (year)	
≤1	4
2–3	14
>3	6
Number of projects and/or committees	
1	5
2–3	11
>3	8

**Table 2:** Characteristics of participating researchers (*n* = 24).

Characteristic	Number
Sex	
Female	15
Male	9
Age (year)	
<40	4
40–64	20
≥65	0
Location of residence	
Western Canada	15
Ontario	8
Quebec	0
Eastern Canada	0
Territories	0
Prefer not to answer	1
Duration in Can‐SOLVE CKD Network (year)	
≤1	1
2–3	6
>3	17
Number of projects and/or committees	
1	16
2–3	7
>3	0

We characterized four overarching themes according to participants' accounts of the dynamic nature of patient engagement within the network (Supporting Information): (1) Reinforcing a shared purpose; (2) Fostering a culture of responsive and innovative research; (3) Aligning priorities, goals and needs and (4) Building a path to sustainability. Participants' initial apprehensions about patient engagement evolved into appreciation of its value and potential impact on kidney health innovation. The concept of a research ‘paradigm shift’ was articulated by one researcher participant (R12) and reflected across most patient and researcher interviews—participants recognized the increasing support for and embeddedness of patient engagement in routine health research practices and were enthusiastic about being on the forefront of this new era. They emphasized how formalized but flexible engagement support was necessary to align network and patient partner research priorities and to develop practical solutions to real‐world problems facing healthcare delivery. Both patients and researchers pointed to a need to anticipate sustainability of network infrastructure, engagement resources and research products. Identified themes and subthemes are described in the following section, with additional exemplar quotes provided in Table 3.

**Table 3:** Additional supporting quotes from patient partners and researchers presented by theme and subtheme.

Patient partner quotes	Researcher quotes
*Theme 1: Reinforcing a shared purpose*	
Subtheme: Learning together	
‘My job has been trying to help everyone figure out what patient engagement looks like, what activities we should be doing, trying to ensure that there is some sort of communication flow’. (P3) ‘It's been a teaching and educational exercise over the last three years’. (P3) ‘Those are all pieces of input that I put in there, and [Researcher‐X] and the team have all really bent over backwards for me, my lack of knowledge and me being really green to this stuff. And I've tried my best, to be honest’. (P7) ‘Patients didn't know what they were getting into, researchers also had no idea what they were getting into, and I think overall there are some things that completely surprised them. And in general, they [researchers] are very impressed by patient engagement and the patients that have been involved’. (P11)	‘I'm just learning and doing as I go, hoping I'm always keeping the patient in the best interest and including them’. (R2) ‘We didn't have any guidelines; we just learned from experience and leveraged our experience working with First Nation community members and adapting some of those philosophies when working with patient partners’. (R3) ‘I figured out just asking them [patient partners]: “Does this work? Doesn't this work? What do you think about this?” It's the best way to do it, it becomes a lot easier’. (R4) ‘In terms of trying to fit a formal structure around it, yes [first time engaging patients]. But we do a bunch of work with the patients particularly who have been participants in our trials in an informal structure’. (R9) ‘I was expecting a little better structure. I think a lot of this has been on the job learning’. (R14)
Subtheme: Collective commitment	
‘When I think of a patient lead or patient‐oriented research, I see the patient at the very centre and their role being clearly defined from the very beginning’. (FG‐P1) ‘I think there's something really opening up, too, in terms of determining direction and choice of research based on what patients are saying. And I think the beginning of patient research from that perspective is truly exciting’. (FG‐P2) ‘The research team really had a patient‐oriented focus and [that was top of mind] … it's more like we're driving the bus or trying to drive the bus’. (P3)	‘Ultimately the end goal is for us to improve kidney health for people living with chronic kidney disease, and so, yes, I think at every stage of the process we value their [patient partner] feedback and we try to incorporate it pretty much all the time’. (R3) ‘I think you have to be open minded, and you have to acknowledge that these people bring a unique perspective and that we're all here to learn something from each other. So, open your mind and learn something from them because they've got a lot to teach’. (R8) ‘When you think about it, how ridiculous it is to have a service industry, which in some ways we are, that completely ignores the input from their customers. [Person‐X] spoke about that at one of the initial Can‐SOLVE meetings, and it was like a penny dropped that this was clearly wrong and that we do need to engage patient partners’. (R13) ‘I think they have to continue to keep us honest, right? What we promised and what we wrote, we have to carry through it’. (R14)
Subtheme: Evolving attitudes	
‘I was surprised that doctors were coming over and tapping me on the shoulder and saying: “I have someone for you.” Doctors have seen the value we bring and have invited us to participate’. (P4) ‘The biggest [surprise], I guess, is that my voice was being recognized, [being] acknowledged’. (P11) ‘I think the researchers need to not be afraid of asking us’. (P12)	‘I actually never really thought I would have patients on my research team, but for me, I think it's new and I learned from them’. (R2) ‘What I've learned is more perspective. I think working with these kids, the biggest advantage has been giving me perspective, as a clinician, where they're coming from. I had no idea five years ago what life was really like for these kids’. (R7) ‘I think there has been a shift for sure, and that [researchers] are starting to shift more. I hear them talking a lot more about including patients … Asking people what they want before we tell them what they want’. (R10)
*Theme 2: Fostering a culture of responsive and innovative research*	
Subtheme: Accessible supports	
‘I usually have a sit‐down meeting once every two weeks, just about business in general and keeping her [other patient partner] up to date on everything’. (P6) ‘Knowing when to pull back or allow space for people to be healthy and to be well, because we don't want to make the people that are involved in the network any more sick or tired than they already are’. (P9) ‘I thought getting to know the other patients was really helpful in terms of sort of understanding what was going on especially the patients that had sort of been there before Can‐SOLVE was officially Can‐SOLVE’. (P12)	‘We find that corresponding by phone individually is very helpful for [patient partners] as well … First a conference call with all the patients and then individual follow‐up with each of them’. (R1) ‘It's taken some work to get us where we are now, but that's not because we don't have the traits, it's just that there had to be some circling and getting to know each other’. (R4) ‘Patients are no different than researchers—they have different priorities, different challenges, different personalities, and they have the challenges posed by their health as well’. (R12)
Subtheme: Strengthened process and product	
‘The researchers were saying, “Well, we can't focus on that because it's phantom.” And we were saying, “It may be phantom to you, but it's not phantom to us. Just because you can't now tell us what's causing it doesn't mean it's not real.” And they started changing their tune’. (P8) ‘[We are trying] to get people the best background information as possible that we can. Same thing with Wabishki Bizhiko Skaanj, we started right from scratch with that one. Like, there was nothing, they just told us, “We need a cultural sensitivity training,” and the slate was clean’. (P10) ‘If we're there in the initial conversations we can help shape [the projects], and something very simple [can have] a profound positive impact on how to present it to patients’. (P12)	‘There's a place for [foundational research], but there's also a big place for applied research that's going to make a difference. And if you want to make a real difference, the first thing you have to do is ask the people whose lives will be impacted, what kind of a difference they want made’. (R10) ‘In the end we're still going to need health research, whether we can find people to buy in or not. And where I come back to you is it is a paradigm shift. It is required. It's going to make the research better, so we have to keep working that way’. (R12) ‘[Patient partner] gave us some very helpful feedback about the reluctance for patients to engage in studies that look promising and yet there's an untreated comparative group. We had to deal with that … by amending the protocol so that if the studies were positive, then patients would be invited for an open‐label extension of that study’. (R13)
*Theme 3: Aligning priorities, goals, and needs*	
Subtheme: Amenability to patient involvement	
‘They [researchers] don't expect enough of us, in general. They try to give us like really simple small things. We've encouraged [Researcher‐X], as part of our project, to like give us more and we'll tell [them] when it's too much’. (FG‐P1) ‘You can see in the teams when the leader is very passionate, the rest of the team follows. But in the ones that are okay, that's kind of good, the patient engagement piece for those projects … kind of like slips through the cracks a bit’. (FG‐P2) ‘In the first two years [we] soft pedaled to give the projects a little bit of time to get up and going and figure out what works and what doesn't. [Now] we are becoming far clearer in regard to the expectation as it relates to patient engagement’. (P3) ‘Our coordinator, has been right there dealing with me, dealing with my lack of technology, and she [is the] the go‐to’. (P7)	‘We're able to kind of take it [patient engagement] to the next level. But I think as a research team who's maybe new to patient engagement, might not have a lot of experience, they might not be as open as we are to being adaptable to patient partner's needs’. (R3) ‘The only other thing that is a bit difficult is that the pace of [research], for a physician and a researcher, is different than the pace of what patient partners [are expecting]’. (R4) ‘I think unfortunately Can‐SOLVE is very adult focused and integrating youth into the adult world doesn't really work. In order to bring these kids and their parents into an upper level of Can‐SOLVE, I think it needs to be something that they can relate to’. (R7) ‘I would say that the type of research we're doing in this project lends itself really well with the patient partnerships at all phases. But I can totally appreciate that some other types of research would not, and that'll be okay too’. (R11)
Subtheme: Mutually productive relationships	
‘[In] the ideal world patients are co‐authors on papers. I have already been a co‐author on several papers [and] that is where we should be going. That's the way research should be thinking of our input. I mean, it's only to their benefit that the patients are [involved]’. (FG‐P1) ‘I [gave] my feedback on whether I think it's beneficial on behalf of the patients I work with. I think of a patient lead or patient oriented research, I see the patient almost at the very center … [When] I go back to my area I want to be able to say to the patients: “This is what your role will be, this is what you'll give back, and this is what you'll get”’. (FG‐P1) ‘My expectations, whatever knowledge I could gain, that I could share with people with kidney disease, and to make an improvement in outcomes for people with kidney failure’. (P7) ‘Hopefully [there will be] less patient engagement [in the future] because there would be less kidney disease’. (P9)	‘I felt like they [patient partners] needed to be heard, for sure … at the time to be really mindful of listening to them, but also making sure that we were staying on track’. (R2) ‘We've been inclusive but judicious with what they're [patient partners] involved with. I think that where they've been involved is very helpful, and where they're not involved it probably wouldn't have change anything in that regard’. (R5) ‘I think it's important for patient partners to realize that their view represents their view, not the global patient view. There are different patients, just like different doctors have different opinions about how a condition should be managed. Different patients also have very different perspectives about what they want to know and how they want to be treated … Early on in Can‐SOLVE, sometimes there was a sense that if you didn't agree with them you were somehow ignoring the patient voice’. (R8) ‘I think that their input into what had been identified as the path of the study was helpful and did change the direction of things. I hope they feel the same way. I'm not sure that the patients have had the same satisfaction from their involvement in the study. It's awkward making that leap from what patients want for their own care and experience to what's the best science project here’. (R16)
Subtheme: Harmonizing expectations	
‘We talk a lot about the learning process, not just for us as patient partners being involved … but it's the growing pains that researchers have to go through as well in terms of what to expect from us’. (FG‐P1) ‘I think [my] responsibilities are so different on every aspect of Can‐SOLVE that I'm involved in. I've learned that that's okay, different things work for different provinces and different groups. I think that sometimes we try and find a “one‐size‐fits‐all” … but what we're finding is that it doesn't work across everything’. (FG‐P2)	‘I think the ideal patient partner is someone who comes to the table and recognizes the importance of their position on the research project and really leverages that position to speak the truth of what they exactly feel … People might not be open to providing [us] with their true feedback. But as a patient partner that's exactly what we're looking for. We want them to tell us exactly how it is’. (R3) ‘We're going to have discussions around these things, about how much time they want to do, [what] they want to give to it, what the work involves, and how they want to approach it’. (R10) ‘What we try to do is be very clear as to what the ask is, what we anticipate the timing is, what the touch points are, and how much commitment it would involve, and we try to be very clear upfront’. (R14)
*Theme 4: Building a path to sustainability*	
Subtheme: Value creation	
‘[Researcher] brought up a really interesting point and it just hit me like a ton of bricks, I thought: “Wow, we're not even really taking good care of our donor.” And all our emphasis has been on the recipient and his care, but not the donor’. (FG‐P2) ‘[It is a] very large task to ask patients to take on the Transplant Ambassador Program in their hospital. Now we saying: “Can you recruit other people and train other people and have this program running really well?” I have a sense of ownership and pride in this program]’. (FG‐P2) ‘Being on this committee means I am reading more [health information] … and now I am not afraid to ask questions’. (P11)	‘I can take any problem in healthcare, and I can apply infinite resources to that problem, and it will never be perfect. So, what we have to do is take a step back and say, how much value are we generating from this particular intervention? My sense is we're applying a lot of resources to patient engagement and no matter how many resources we apply, it likely won't be enough. I think what you're going to see is the pendulum swinging back the other way a little bit’. (R5) ‘The purpose of this is still science, right? It is the best science working with our patient partners, but also we have to have a results phase, and these entities have to be productivity and research driven. And that's also what I hear from our patient partners is, “Are we sticking to our timelines? Are we getting results?”’ (R14)
Subtheme: Capacity building	
‘It is a very large task to ask patients to take on the Transplant Ambassador Program in their hospital. Not only are we saying, “This is your project, your baby, manage it,” but also “Can you recruit other people and train other people and have this program running really well?”’ (FG‐P2) ‘I'm just helping to move us along. Everybody needs to be articulate and know about Wabishki Bizhiko Skaanj, and we have some tools to help you … Everybody on the project should really be up to speed on what's happening and move forward that way’. (P10)	‘I would consider it as part of my postdoc training because it is the way of research now. That is something essential that I need to develop, and if I want to have more impactful research, I really do need to engage patients’. (R6) ‘We're pretty good at project management type stuff and we might be good at getting the people together into the room. But then, step back a little bit and move other people forward. Stop trying to be the one who says we have to develop this thing in this way’. (R10)
Subtheme: Sustaining knowledge use	
‘In a perfect world we'll have all 18 projects completed and papers published and the knowledge translation from the projects will very quickly get out there in the world. That work is really a lot of blood, sweat and tears, and I'd hate to see it just put in a box. I hope it's the basis for kidney research going forward’. (P5) ‘I focused my interest on the knowledge translation piece because it was something that really interested me. I didn't really know a lot about it, but the idea of translating information into a way that lay people could understand really interested me. I have had a lot of opportunities to do speaking engagements’. (P12)	‘[We] got a lot of feedback from both researchers as well as the patients on the implementation process. It's hard for them to maybe talk about the technical side of things, but their voices are heard in the sense of the technicality of things … That's the whole point of this, to make it so that when you go to practice it becomes part of your routine care for the patient’. (R2) ‘Very few studies now come with these blockbuster results, and I think it's important then to be able to put it in language that is clear, that the information is written so that people can understand it, and then see how they can integrate this into their care’. (R14)

*Note*: Participant identifiers are indicated in parentheses following each quote. Those beginning with P denote patient participants, those beginning with R denote researcher participants, and FG refers to focus groups.

### 3.1 Reinforcing a shared purpose

Despite having little experience in patient engagement before their joining the network, nearly all patient and researcher participants described approaching their partnerships with an open mind and eagerness to work together to advance the research. The network's clear mandate, strong leadership and shared commitment to patient engagement formed the necessary foundation upon which research partnerships could flourish.

#### 3.1.1 Learning together

Participants reported varying levels of experience and comfort with patient‐oriented research before the network's inception. Although several patient partners and researchers had contributed to early network planning and its funding proposal, they described being underprepared for collaborating subsequently within network project teams. Some researchers drew on prior experiences working with patient advisory groups outside of Can‐SOLVE CKD, and others expressed initial apprehension and dismay over a lack of guidance on patient engagement specific to their projects. Most patient partners described having little to no prior research experience other than as a research participant, and participants outlined a co‐learning exercise as they developed processes together and formalized collaborative engagement structures. As one patient said,

We were falling into the trap of wanting to do too much because we were enthusiastic, but we had to find that balance of what we could conceivably do with our time as volunteers and what was better done by people who are staff. I think in the first year, we always joked about building the plane as we're flying it. (P12)

#### 3.1.2 Collective Commitment

Despite initial apprehension about how to collaborate in research, researchers identified a strong rationale for patient engagement from their projects' outset. They indicated that research engagement could enable realistic, honest and pragmatic discourse about end‐user needs and outlined a commitment to ensure their work resonated with patient partners. Patient partners described a variety of motives for engaging in the network (e.g., ‘build skills’, ‘leave a legacy’, ‘obligation’ to community) but indicated it was critical first to understand why their involvement was needed and how they could best contribute. They explained how mutual appreciation of each other's roles paved the way for confident, prepared and productive research and a patient leadership model where, as one patient explained, ‘we're driving the bus’ (P3).

#### 3.1.3 Evolving attitudes

Several patient partners observed that researchers seemed uncertain initially about how to involve them in the research and hesitant about approaching them with research‐related questions or requests. Although many patients described being uncertain themselves, they expressed an openness to being approached about collaborative problem solving. They indicated these partnerships were critical to move research forward and mitigate concerns about patient engagement pitfalls, such as tokenism. As relationships developed and confidence grew, patient partners described an increasing appreciation of having their voices recognized, and researchers described gaining a grounded perspective of the implications of their work to those living with kidney disease. This translated into growing receptivity to patient engagement among researchers and perceived value in better understanding patients' lived experiences. As one nonclinician researcher described,

I don't see any of these individuals [in a clinical setting], so I can't really understand what they're going through. I think this really humanizes the work … It's really great to get that patient perspective and it's also extremely motivating when you hear these individual stories. (R6)

### 3.2 Fostering a culture of responsive and innovative research

The Can‐SOLVE CKD Network's culture of openness and responsiveness to the needs of diverse projects and members was highlighted across nearly all interviews and focus groups. Patient partners described increasing comfort and confidence with collaborating in research as a result of the formal (e.g., tools, guidance documents, workshops) and informal (e.g., peer mentorship) support structures that evolved over time. Participants appreciated the network's emphasis on patient engagement as a way of making research more meaningful and achieving greater impact from the resulting health innovations.

#### 3.2.1 Accessible supports

Participants acknowledged the network's flexibility and responsiveness to patient partners' needs, which varied across individuals and over time. This included recognizing patient partners as people first, with responsibilities and priorities outside of their network commitments. Researchers described adapting research processes to accommodate patient partners (e.g., technical assistance, conducive scheduling) and remaining flexible when unforeseen obstacles arose, such as patient partner illness. Patient participants related the network's responsiveness to its nonhierarchical structure and emphasis on equity and inclusion among its membership.

Many researchers and patient partners turned to internal network resources to inform their engagement strategies, which matured over the network's term to offer practical, accessible guidance in areas including knowledge translation, engagement competencies and cultural safety. Several researchers had also accessed resources outside of the network (e.g., provincial SPOR resources), but few described connecting with other network teams for support. In contrast, nearly all patient partners articulated the importance of peer mentorship and supported one another informally in the unique experience of partnering in kidney health research. One researcher commended a patient partner for serving as a key support for others on their research team,

She [patient] was enabling all the partnerships, and not only enabling them but ensuring everything was being done appropriately, serving as a resource to patients when they had questions. (R11)

#### 3.2.2 Strengthened process and product

Researchers anticipated that improved research processes resulting from patient engagement would strengthen the resulting health innovation. They provided several examples of how patient partner involvement enhanced patient‐facing materials, interview question wording and outcome selection. A few researchers discussed how ethical issues about trial design raised by patient partners, such as randomization and untreated comparator groups, influenced how they framed research methodology to participants. Researchers anticipated enhanced applicability of health innovations developed in partnership with patients, and patients observed how even seemingly small changes to research approaches can influence outcomes. Patient partners explained how a movement toward greater patient leadership prompted establishment of several core network resources and community engagement strategies (e.g., Indigenous health research learning pathway).^28^ One patient who contributed to both research and core committee activities focused on Indigenous health explained,

I think that's one way that patient partners can help change the way research is done—the way we're engaging with communities, especially Indigenous communities. For me, it's an important thing. (P9)

### 3.3 Aligning priorities, goals and needs

With the diversity of researchers, patient partners and initiatives across the network, participants described how alignment between individual and research‐related goals, priorities and needs was critical for the network's success. Although several patients initially questioned how to best apply their experience and expertise to the various initiatives, expectations surrounding patient engagement became clearer as the research progressed and comfort and familiarity with one another grew. Researchers described being attuned to patients' obligations and priorities and striving to ensure their collaboration remained mutually rewarding, respectful and enjoyable.

#### 3.3.1 Amenability to patient involvement

Initially, researchers and patient partners wondered whether certain project types might be more amenable to patient engagement than others. For example, researchers wondered if projects that were more slowly paced, biomedical or with inexperienced teams may struggle with effective patient involvement. However, patient partners cited research teams' commitment, and responsiveness as key elements supporting engagement regardless of project type. As one basic scientist explained, ‘There is no need for a science background that is particularly advanced’ for patients to contribute to biomedical research (R13); technical concepts could be relayed in a straightforward way, and patients could contribute to other important, nontechnical aspects of research design. Patient partners described relying on communication and trusting relationships to overcome hurdles to engaging in less clinically focused projects. Some researchers and patient partners identified challenges to integrating paediatric patient partners into larger network circles, which they related to a lack of supports to meet the distinct engagement needs of this population.

#### 3.3.2 Mutually productive relationships

Researchers discussed difficulties balancing patient engagement with their research objectives and milestones. Participants relayed infrequent instances where researchers and patient partners had competing agendas or ideas regarding their project's direction. Awareness of each other's needs grew through increased familiarity among the team, habituation to research processes and mechanisms for revisiting priorities. Researchers described being mindful of patient partners' other commitments and trying to limit their requests for involvement to areas of highest yield for both parties. Patient partners reinforced that for them to contribute most effectively, all parties needed to be aligned in their vision and goals. Patient partners suggested that acknowledgement of their contributions to the research, such as through co‐authorship on manuscripts, would foster trust and a desire to collaborate on future research initiatives.

#### 3.3.3 Harmonizing expectations

Patient partners and researchers across most projects described having open conversations about project goals initially, whereas more systematic processes to establish expectations came later with a better understanding of team members' needs and project direction. Patient partners acknowledged the diversity in network projects and membership and rejected a singular approach to engagement. As one patient said during a focus group,

Different things work for different provinces and different groups. I think that sometimes we try and find a one‐size‐fits‐all [approach] … but what we're finding is that it doesn't work across everything. (FG‐P2)

Some researchers described tempering initial high expectations for prompt, observable changes to their research and growing to appreciate other benefits of research partnerships, including community connection, honest feedback and compassion rooted in lived experience.

### 3.4 Building a path to sustainability

Researcher and patient participants described how sustainability of the network's research, infrastructure and patient engagement had been a priority for the network from the outset. They conveyed a sense of pride and ownership of the network's achievements, and indicated it was a shared responsibility of network membership to ensure the collaborative relationships and best engagement practices continued beyond the network's funding term. Key drivers of sustainability identified across researcher and patient partner interviews included demonstrating value of the research arising from patient engagement, building capacity for research engagement through skills development and hands‐on learning, and supporting the uptake, or implementation, of research generated by the network in real‐world settings.

#### 3.4.1 Value creation

Researchers explained how systemic changes to research engaging patients must demonstrate not just impact, but value for them to be sustainable long‐term. Few questioned whether the potential impact of patient‐oriented research was enough to justify continued high costs, resources and human capital. However, others re‐iterated the tangible and/or anticipated benefits to health innovation, healthcare delivery and patient outcomes to justify its longevity. Several patient partners noted personal impacts of their involvement in Can‐SOLVE CKD, such as enhanced disease‐related knowledge, research skills and confidence during research and clinical encounters. Participants explained how continued awareness in public and research circles was needed to sustain patient engagement beyond the network's funding term and dedicated patient‐oriented research funding opportunities. One researcher expressed the following concerns,

It [patient‐oriented research] could be just another [funding] program that comes to an end … If they're able to get a bit more momentum behind the public to speak to this—by public, I mean members of the public but also voters, because it's the minister of health that ultimately will decide on these issues—then I think that will help with the sustainability of it. (R12)

#### 3.4.2 Capacity building

Patients and researchers explained how early identified gaps in support provided opportunities to develop internal resources that could enhance and sustain patient‐oriented research capacity. Patient partners described how resources that were under development at the time of interviews (e.g., communities of practice, Indigenous health research learning pathways)^29^ were expected to promote research skills development and sustain culturally safe research practices. Building on the strong bonds formed among patient partners within the network, they discussed formalizing peer‐led training of patient partners in the how‐to of patient leadership. One researcher observed colleagues becoming increasingly comfortable with patient engagement as ‘the way of [doing] research now’ (R6), and others described how principles of collaborative research were being integrated with their institutions' graduate and postgraduate research training programmes. For established researchers and trainees, most patient engagement training took place through hands‐on, experiential learning and were supplemented by network tools and resources.

#### 3.4.3 Sustaining knowledge use

Patient partners and researchers indicated that moving the knowledge generated through network activities into the applied setting was critical to sustain use of health innovations over time. They discussed the importance of considering real‐world implementation and evaluation at the outset of their projects, and aligning their patient engagement approaches with this goal of relevant, usable research. Patient partners discussed being naturally attracted to areas where they could advance research application and observe changes in kidney care and patient outcomes on a larger scale. Both researchers and patients outlined how patient partners had contributed to traditional, academic strategies for sharing research knowledge (e.g., co‐authorship on published reports) as well as community engagement activities to share tools and personal narratives related to network initiatives. Both researchers and patient partners described a commitment to seeing their initiatives through to completion, and suggested that patient engagement was critical for promoting use of research in clinical practice or communities affected by kidney disease. One researcher remarked,

The other big thing [is] implementation and dissemination. We develop this tool, but how do we ensure uptake? Patient input is the key component. (R1)

## 4 DISCUSSION

In this study, we provided a real‐world example of the dynamic nature of engaging patients as research partners in a national kidney health research network. In exploring perspectives across a wide variety of network activities, we identified several themes capturing the adaptive processes and growing confidence of the network to integrate patients as partners. Mutual appreciation of the purpose of patient‐oriented research formed the foundation upon which patient partners and researchers aligned their priorities and moved projects forward. In response to identified gaps in initial engagement guidance offered by the network, members co‐developed internal resources and processes that formalized a concerted, inclusive approach to partnership across initiatives. This promoted greater transparency and accountability and helped align the expectations of both researchers and patient partners. The network's emphasis on impactful, applicable research from the outset encouraged participants to think ahead to how network infrastructure, health innovations and patient engagement could be sustained long‐term.

Several frameworks exist in the academic literature to guide patient engagement in research,^13^, ^15^, ^16^, ^30^, ^31^ although their application in the context of a diverse network like Can‐SOLVE CKD has not been clearly defined. Recognizing that high‐level frameworks leave room for customization and flexibility; our findings support the approach taken by network members of adapting existing guidance and developing novel strategies to support engagement where gaps were identified. These findings align with those of Greenhalgh et al.,^15^ who assert that a single, off‐the‐shelf framework may be less useful than a menu of evidence‐based resources. This opportunity to customize engagement approaches based on network members' needs led to the creation of several tools and products to support research processes targeted to patient and researcher audiences alike.^28^, ^29^, ^32^, ^33^ The Can‐SOLVE CKD Network is not unique in its application of guiding frameworks for patient engagement and commitment to key principles of inclusivity, support and mutual trust.^34^, ^35^, ^36^, ^37^ Whereas others have reported similar challenges to partner with patients in research, such as avoiding tokenism and balancing individual needs with research objectives,^17^ our study is among the first to describe how research practices, supports and perspectives adapted to new and unforeseen issues arising over the course of a large‐scale network partnership. In one qualitative study of researchers' and patients' experiences in a patient‐oriented research programme centred on case management, participants described strengthened relationships over time, the importance of strong structural supports and balancing diverse interests.^38^ Our study extends and enhances these findings through its emphasis on the dynamic nature of network partnerships, responsive leadership and implications for sustainability across a broad scope of research projects and network initiatives.

The network environment was a key success factor, specifically its emphasis on relationship‐building across initiatives. Initially, much time was devoted to establishing interpersonal connections, devising ways to collaborate effectively and overcoming steep learning curves for both patients and researchers. This emphasis on relationship building reflects a growth mindset,^39^ whereby researchers and patient partners worked in concert to first determine their specific projects' needs and then built upon their experiential knowledge toward relevant, usable research products. This growth occurred to a large extent organically alongside increasing confidence, competency and trust among network members. Consistent with our findings, other reports have emphasized cohesive relationships and positive team interactions as a way of facilitating patient engagement and ensuring meaningful engagement experiences.^16^, ^40^, ^41^, ^42^ In response to the variability in Can‐SOLVE CKD project needs and patient partners' commitment to improving engagement practices, the network developed internal resources with membership input over time that created more structure and expectations around patient engagement (see Toolkit for Project Leads, available at https://cansolveckd.ca/resources/). These supports matured over time, enabled teams to tackle challenges as they arose and fostered a culture of responsiveness and innovation as the network's capacity grew.

Recognizing the significant financial and human costs of engaging patients in a national research network, participants described how impactful interventions and measurable outputs would be critical for sustaining the network and patient engagement more broadly. As participants reflected on their journey, pockets of innovation were highlighted, including patient leadership in research projects (e.g., Transplant Ambassador Programme),^43^ knowledge mobilization and strategies for equity, diversity and inclusion. One example that featured prominently in interviews was the Wabishki Bizhiko Skaanj learning pathway,^28^ which was developed to address identified gaps in Indigenous health research competence in the network. The opportunity to leverage strong connections between patient partners across the network could further enable sustainable research engagement and capacity, whereby patient partners with varied research and kidney disease lived experience could assist, support and train one another using principles of peer mentorship.

Our findings raise important questions about the next steps for the network and generated research knowledge upon completion of the funding term. As most network projects focused on developing and testing strategies to address identified gaps in kidney care delivery,^14^ several participants questioned how the resulting research would make its way into practice to influence healthcare delivery and patient outcomes. With the increasing adoption of collaborative models of health research, opportunities to engage patients in the dissemination and application of research are emerging.^33^, ^44^ As patients will ultimately use or benefit from the output of health research activities, their engagement in research should not necessarily cease once a study has concluded or a health intervention has been generated—continued engagement in identifying barriers and facilitators to implementation, tailoring interventions and evaluating outcomes are critical for promoting uptake and sustainability in different contexts.^44^ Moreover, building capacity in the know‐how of patient engagement among trainee and early‐career researchers can foster a ‘business as usual’ attitude toward research partnerships that translate to real‐world applications.^45^ ^,p.4^ These issues require further study, practice and adaptation as appropriate to promote sustainability of network initiatives.

Many of the challenges to patient engagement described by Bird et al.,^40^ such as power imbalances, were overcome by the supportive research environment. In ensuring patient embeddedness across all network activities and access to capacity‐building resources, patient partners' and researchers' initial scepticism gave way to new skills, enhanced confidence and tangible research outputs over time.^46^, ^47^, ^48^ Strong relationships coupled with shared purpose largely averted tokenism and its consequences, such as poor engagement experiences and lack of demonstrable impact.^49^ However, engagement success relies on many factors, some of which are outside the control of research teams.^50^, ^51^ The varying research skills and engagement experience of participants meant that additional resources were needed to address early challenges to research collaboration. Also, the vast scope of research disciplines and project foci within the network necessitated tailored approaches to supporting each team's unique needs, which were not always possible given resource and capacity constraints. As a result, some communities may have been unintentionally less well supported, such as paediatric patient and caregiver partners for whom the network's ‘adult‐focused’ engagement structures may not be conducive. Several barriers to diversity within patient‐oriented research have been identified, such as lower literacy, communication challenges and socioeconomic disadvantage, which can lead to the underrepresentation of certain groups.^52^ Recognition of the diversity of individuals engaging as research partners is a critical first step toward tailoring engagement infrastructure and processes to meet their needs and those of the research. Going forward, the network has prioritized an equitable and inclusive approach to its research activities and patient partnerships that includes guidance on culturally safe health research practices, considerations for sex‐/gender‐based and paediatric research engagement, resources to build capacity and confidence in research engagement, and mechanisms to communicate concerns and address challenges as they arise.

The main strength of our study lies in its capture of diverse perspectives across a national patient‐oriented research network, although we acknowledge some limitations. We did not attempt repeated data collection from participants over time, which may have enabled the refinement of concepts related to change or evolution. However, our staggered recruitment approach over the later network stages captured nuances in how patient engagement practices adapted and resulted in tangible products. Perspectives were limited to those who agreed to participate, which for focus groups meant individuals present at the in‐person annual network meeting. Invitations for phone or virtual interviews were extended to all network members and aimed to reduce barriers to participation for all interested individuals. Given the close relationships among network members that our study highlighted, it is possible that participants tempered their responses or presented overly optimistic assessments of their engagement experiences. To address this, a research team member with whom participants were unfamiliar led interviews/focus groups and gave assurances of confidentiality. Lastly, this study was undertaken exclusively with Can‐SOLVE CKD Network members, although we anticipate that our broad characterization of findings will be transferrable to both large‐scale, patient‐oriented research contexts and individual teams seeking guidance on patient engagement.

## 5 CONCLUSION

Across a diverse, national patient‐oriented kidney health research network, approaches and attitudes toward patient engagement evolved over time as relationships solidified, confidences grew and priorities became better aligned. Optimizing support structures was a driver of effective engagement and took place both through the adaptation of existing resources and the development of new ones to address identified gaps. The sustainability of the network's innovations and patient partnerships was emphasized across participants and highlights the need for future work to examine the impact and value of network products and explore avenues for expanding patient‐engaged kidney health research capacity.

## AUTHOR CONTRIBUTIONS

Meghan J. Elliott and Nicolas Fernandez conceived of this study, participated in its co‐design, conducted interviews/focus groups, data analysis and drafted the manuscript. Tamara L. McCarron participated in data analysis, results interpretation and manuscript preparation. Kara Schick‐Makaroff contributed to the study design and provided methods support, including qualitative data analysis and interpretation. Leah Getchell assisted with participant recruitment, data collection during focus groups and interpretation of findings. Braden Manns contributed to the study design and provided operational support and mentorship to the research team. All authors provided a critical review of the manuscript and approved the final version.

## CONFLICT OF INTEREST STATEMENT

The authors declare no conflicts of interest.

## ETHICS STATEMENT

This research project was approved by the Conjoint Health Research Ethics Board (CHREB) at the University of Calgary (REB18‐0131) and the Comité d'éthique de la recherche en santé (CÉRES) at the University of Montreal (#17‐138‐CERES‐D). All participants provided informed consent for participation in this study.

## Supporting information

Supporting information.

## Data Availability

The data sets used and/or analysed during the current study have not been made publicly available due to the potential identifiability of participants from transcript data. Data may be available upon reasonable request to the corresponding author.
