# Supplementary material for: The dynamic nature of patient engagement within a Canadian patient‐oriented kidney health research network: Perspectives of researchers and patient partners
Source: Health Expect. 2023 Jan 27;26(2):905–18. doi: 10.1111/hex.13716 (PMC10010076; doi:10.1111/hex.13716)
Supplement: Supplementary file 1 — Supporting information. [file HEX-26--s001.docx]

**Supplemental material**

**Focus group topic guide**

| **Focus group discussion** |
| --- |
| **Broad introductory question**  Please tell us about your involvement in Can-SOLVE CKD (i.e. projects, committees, councils). |
| **Engagement within Can-SOLVE CKD**  What are the responsibilities of patients within your research project/committee? What tasks have they undertaken?  How were these responsibilities determined within your research project/committee?  What are some challenges you have identified with working together with patients and researchers in your project(s), if any? |
| **Expectations for engagement**  When starting out, what were your expectations for what researchers should bring to your Can-SOLVE CKD research team? What do you see as the actual contributions of the researchers to your research team?  When starting out, what were your expectations for what patient partners should bring to your Can-SOLVE CKD research team? What do you see as the actual contributions of the patient partners to your research team?  How do these views and expectations compare to before you started working within Can-SOLVE CKD? Have your views changed? If yes, explain.  Do you think that the roles of the patients and researchers in your project have changed over the past 2-3 years? Explain. |
| **Characterizing ideal engagement**  How would you describe the ideal patient partner for kidney health research? For your project specifically?  How would you describe the ideal researcher partner for kidney health research? For your project specifically?  Tell us how you see patient engagement in kidney health research changing/evolving in the next 5 years? Specifically, within Can-SOLVE CKD? |
| **Wrap up** |
| We have come to the end of our session today. What we have heard is:______________.  Are there any other comments you would like to add? |

**Interview question guide**

| **Engagement within Can-SOLVE CKD** |
| --- |
| Please tell us about your involvement in Can-SOLVE CKD (i.e. projects, committees, councils). What are your responsibilities within your research project(s)/committee(s)? |
| What are the responsibilities of patients within your research project/committee? What tasks have they undertaken? |
| How were these responsibilities determined within your research project/committee? |
| What are some challenges you have identified with working together with patients and researchers in your project(s), if any? |
| **Expectations for engagement** |
| When starting out, what were your expectations for what researchers should bring to your Can-SOLVE CKD research team? What do you see as the actual contributions of the researchers to your research team? |
| When starting out, what were your expectations for what patient partners should bring to your Can-SOLVE CKD research team? What do you see as the actual contributions of the patient partners to your research team? |
| How do these views and expectations compare to before you started working within Can-SOLVE CKD? Have your views changed? If yes, explain. |
| In what ways have the roles of the patients and researchers in your project changed over the past 2-3 years? Explain. |
| **Characterizing ideal engagement** |
| How would you describe the ideal patient partner for kidney health research? For your project specifically? |
| How would you describe the ideal researcher partner for kidney health research? For your project specifically? |
| Tell us how you see patient engagement in kidney health research changing/evolving in the next 5 years? Specifically, within Can-SOLVE CKD? |
| **Wrap-up** |
| Are there any other comments you would like to add? |

**Supplemental figure.** Schematic depicting the relationship between thematic findings

**
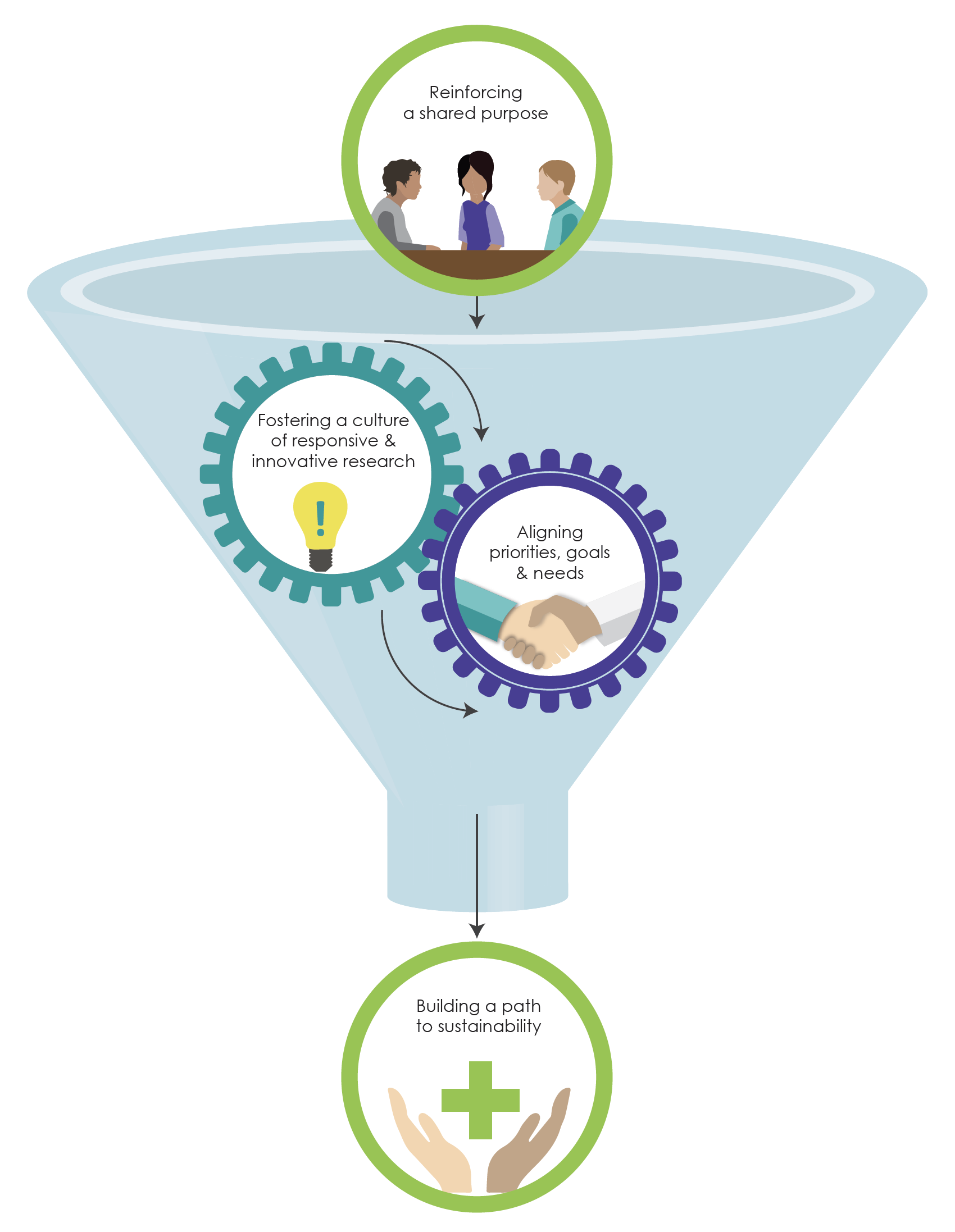
**
